# 11,20-Dihy­droxy-3-oxopregna-1,4-dien-21-oic acid monohydrate

**DOI:** 10.1107/S1600536811015224

**Published:** 2011-05-07

**Authors:** Zhanqiang Guo, Shengan Tang, Hongquan Duan

**Affiliations:** a School of Pharmaceutical Sciences, Research Center of Basic Medical Sciences, Tianjin Medical University, Tianjin 300070, People’s Republic of China

## Abstract

The title compound, C_21_H_28_O_5_·H_2_O, is the hydrate of a steroid derivative and was obtained by degradation of solid prednisolone sodium phosphate. The six C atoms in ring *A* are nearly co-planar with a mean deviation of 0.015 Å. Rings *B* and *C* are both in chair conformations, while ring *D* has an envelope form. In the crystal, inter­molecular O—H⋯O hydrogen-bonding inter­actions occur between the hy­droxy groups, carbonyl O atoms and solvent water mol­ecules, resulting in an overall three-dimensional structure.

## Related literature

For general background to substances related to prednisolone sodium phosphate, see: Dekker (1980 ▶); Stroud *et al.* (1980 ▶); Mason (1938 ▶); Edmonds *et al.* (2006 ▶); Gazdag *et al.* (1998 ▶). For related structures, see: Suitschmezian *et al.* (2008 ▶); Rachwal *et al.* (1996 ▶). 
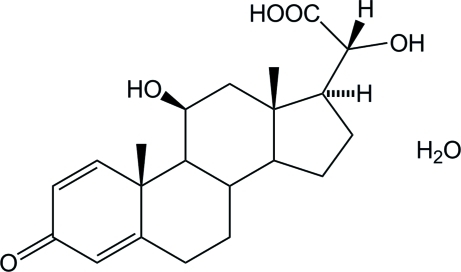


## Experimental

### 

#### Crystal data

C_21_H_28_O_5_·H_2_O
*M* *_r_* = 378.45
Orthorhombic, 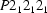

*a* = 11.801 (2) Å
*b* = 12.526 (3) Å
*c* = 12.884 (3) Å
*V* = 1904.5 (7) Å^3^
*Z* = 4
Mo *K*α radiation
μ = 0.10 mm^−1^
*T* = 113 K
0.20 × 0.10 × 0.10 mm

#### Data collection

Rigaku Saturn CCD area-detector diffractometer
Absorption correction: ψ scan (*CrystalClear*; Rigaku, 2005 ▶) *T*_min_ = 0.981, *T*_max_ = 0.991
13023 measured reflections
1922 independent reflections
1809 reflections with *I* > 2σ(*I*)
*R*_int_ = 0.046

#### Refinement

*R*[*F* ^2^ > 2σ(*F* ^2^)] = 0.035
*wR*(*F* ^2^) = 0.085
*S* = 1.06
1922 reflections
232 parameters
H-atom parameters constrained
Δρ_max_ = 0.22 e Å^−3^
Δρ_min_ = −0.18 e Å^−3^

### 

Data collection: *CrystalClear* (Rigaku, 2005 ▶); cell refinement: *CrystalClear*; data reduction: *CrystalClear*; program(s) used to solve structure: *SHELXS97* (Sheldrick, 2008 ▶); program(s) used to refine structure: *SHELXL97* (Sheldrick, 2008 ▶); molecular graphics: *SHELXTL* (Sheldrick, 2008 ▶); software used to prepare material for publication: *SHELXTL*.

## Supplementary Material

Crystal structure: contains datablocks I, global. DOI: 10.1107/S1600536811015224/bh2337sup1.cif

Structure factors: contains datablocks I. DOI: 10.1107/S1600536811015224/bh2337Isup2.hkl

Additional supplementary materials:  crystallographic information; 3D view; checkCIF report

## Figures and Tables

**Table 1:** Hydrogen-bond geometry (Å, °)

*D*—H⋯*A*	*D*—H	H⋯*A*	*D*⋯*A*	*D*—H⋯*A*
O2—H2⋯O1^i^	0.82	1.92	2.708 (2)	161
O3—H3⋯O2^ii^	0.82	2.06	2.819 (2)	153
O4—H4⋯O6^iii^	0.82	1.84	2.646 (2)	167
O6—H61⋯O1	0.86	1.92	2.765 (2)	165
O6—H62⋯O5^iv^	0.86	2.10	2.938 (2)	166

Symmetry codes: (i) 
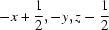
; (ii) 
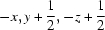
; (iii) 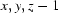; (iv) 
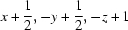
.

**Fig. 1.**
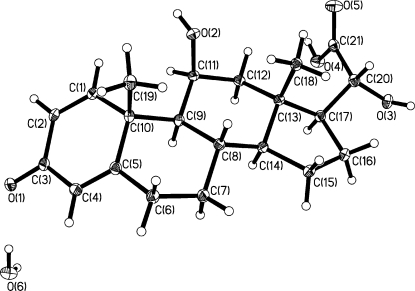
The molecular structure of (I), with atom labels and 30% probability displacement ellipsoids, and H atoms are shown as small spheres of arbitrary radius.

**Fig. 2.**
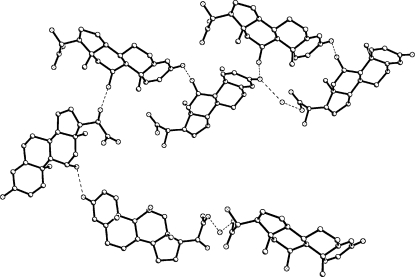
The packing of (I), showing the three-dimensional structure, with intermolecular hydrogen bonds (dashed lines); for clarity H atoms have been omitted.

## supplementary crystallographic information

### Comment

In the molecule of the title compound, (Fig. 1), all bond lengths and angles are within normal ranges (Mason, 1938; Edmonds *et al.*, 2006; Gazdag *et al.*, 1998; Rachwal *et al.*, 1996). Six carbon atoms in ring *A* (C1···C5/C10) are nearly in the same plane with the average atomic displacement of 0.015 Å. Rings *B* (C5···C10) and *C* (C8/C9/C11···C14) are both in chair conformations. Ring *D* (C13···C17) has an envelope form with C13 as the out-of-plane atom. Through extensive O—H···O hydrogen bonds between the main molecule and lattice water molecule, a three dimensional supramolecular network is formed. The water molecules are involved in O—H···O hydrogen bonding with atoms O1, O5 and O4 belonging to hydroxy groups, and intermolecular O—H···O hydrogen-bonding interactions are formed between hydroxy groups and carbonyl O atom, resulting in an overall three-dimensional crystal structure (Fig. 2).

### Experimental

The title compound (Dekker, 1980; Stroud *et al.*, 1980) was obtained by degradation of solid prednisolone sodium phosphate at 373 K for 72 h, then extracted and isolated using HSCCC followed by preparative HPLC. Finally, the crystals were prepared by slow evaporation of the solvent from a saturated solution in methanol/acetone/H_2_O at room temperature (Suitschmezian *et al.*, 2008).

### Refinement

H atoms attached to carbons were placed at calculated positions with C—H = 0.93 Å (aromatic) or 0.96–0.98 Å (*sp*^3^ C-atom). H atoms attached to oxygen was located in difference maps. All H atoms were refined in the riding-model approximation with isotropic displacement parameters (set at 1.2–1.5 times of the *U*_eq_ of the parent atom). As the structure has no significant anomalous dispersion, the Friedel-pair reflections (1436) were merged and the absolute configuration was assumed from synthesis.

### Crystal data

**Table:**

C_21_H_28_O_5_·H_2_O	*F*(000) = 816
*M**_r_* = 378.45	*D*_x_ = 1.320 Mg m^−^^3^
Orthorhombic, *P*2_1_2_1_2_1_	Mo *K*α radiation, λ = 0.71073 Å
Hall symbol: P 2ac 2ab	Cell parameters from 25 reflections
*a* = 11.801 (2) Å	θ = 4.1–22.5°
*b* = 12.526 (3) Å	µ = 0.10 mm^−^^1^
*c* = 12.884 (3) Å	*T* = 113 K
*V* = 1904.5 (7) Å^3^	Block, colourless
*Z* = 4	0.20 × 0.10 × 0.10 mm

### Data collection

**Table:**

Rigaku Saturn CCD area=detector diffractometer	1922 independent reflections
Radiation source: rotating anode	1809 reflections with *I* > 2σ(*I*)
confocal	*R*_int_ = 0.046
Detector resolution: 7.31 pixels mm^-1^	θ_max_ = 25.0°, θ_min_ = 2.3°
ω and φ scans	*h* = −12→14
Absorption correction: ψ scan (*CrystalClear*; Rigaku, 2005)	*k* = −14→11
*T*_min_ = 0.981, *T*_max_ = 0.991	*l* = −15→15
13023 measured reflections	

### Refinement

**Table:**

Refinement on *F*^2^	Secondary atom site location: difference Fourier map
Least-squares matrix: full	Hydrogen site location: inferred from neighbouring sites
*R*[*F*^2^ > 2σ(*F*^2^)] = 0.035	H-atom parameters constrained
*wR*(*F*^2^) = 0.085	*w* = 1/[σ^2^(*F*_o_^2^) + (0.0589*P*)^2^ + 0.0073*P*] where *P* = (*F*_o_^2^ + 2*F*_c_^2^)/3
*S* = 1.06	(Δ/σ)_max_ = 0.001
1922 reflections	Δρ_max_ = 0.22 e Å^−^^3^
232 parameters	Δρ_min_ = −0.17 e Å^−^^3^
0 restraints	Extinction correction: *SHELXL97* (Sheldrick, 2008), Fc^*^=kFc[1+0.001xFc^2^λ^3^/sin(2θ)]^-1/4^
0 constraints	Extinction coefficient: 0.026 (3)
Primary atom site location: structure-invariant direct methods	

### Fractional atomic coordinates and isotropic or equivalent isotropic displacement parameters (Å^2^)

**Table:**

	*x*	*y*	*z*	*U*_iso_*/*U*_eq_	
O1	0.30262 (15)	0.07528 (11)	0.89120 (11)	0.0247 (4)	
O2	0.06804 (6)	0.10188 (5)	0.41310 (5)	0.0210 (4)	
H2	0.0958	0.0443	0.3967	0.031*	
O3	0.15678 (6)	0.53818 (5)	0.12731 (5)	0.0257 (4)	
H3	0.1016	0.5765	0.1169	0.039*	
O4	0.29729 (6)	0.36129 (5)	0.13021 (5)	0.0287 (4)	
H4	0.3291	0.3118	0.1004	0.043*	
O5	0.14172 (6)	0.27905 (5)	0.07258 (5)	0.0340 (4)	
O6	0.41158 (6)	0.22413 (5)	1.01436 (5)	0.0318 (5)	
H61	0.3671	0.1799	0.9832	0.048*	
H62	0.4755	0.2338	0.9842	0.048*	
C1	0.17278 (6)	0.04907 (5)	0.64360 (5)	0.0194 (5)	
H1A	0.1791	0.0060	0.5851	0.023*	
C2	0.2366 (2)	0.02545 (16)	0.72564 (16)	0.0199 (5)	
H2A	0.2822	−0.0351	0.7241	0.024*	
C3	0.2363 (2)	0.09259 (17)	0.81752 (17)	0.0207 (5)	
C4	0.1569 (2)	0.18171 (17)	0.82009 (17)	0.0224 (5)	
H4A	0.1530	0.2240	0.8793	0.027*	
C5	0.0899 (2)	0.20402 (17)	0.73978 (16)	0.0201 (5)	
C6	0.0106 (2)	0.29859 (16)	0.74117 (17)	0.0241 (5)	
H6A	0.0172	0.3356	0.8070	0.029*	
H6B	−0.0670	0.2740	0.7340	0.029*	
C7	0.0390 (2)	0.37501 (17)	0.65283 (16)	0.0228 (5)	
H7A	0.1116	0.4084	0.6669	0.027*	
H7B	−0.0178	0.4309	0.6503	0.027*	
C8	0.0444 (2)	0.31972 (16)	0.54711 (16)	0.0181 (5)	
H8A	−0.0314	0.2940	0.5284	0.022*	
C9	0.12720 (19)	0.22354 (16)	0.55283 (15)	0.0167 (5)	
H9A	0.1988	0.2540	0.5775	0.020*	
C10	0.09127 (19)	0.14101 (16)	0.63966 (16)	0.0184 (5)	
C11	0.15618 (19)	0.17277 (15)	0.44709 (16)	0.0174 (5)	
H11A	0.2245	0.1295	0.4572	0.021*	
C12	0.18324 (19)	0.25475 (16)	0.36265 (16)	0.0181 (5)	
H12A	0.1871	0.2185	0.2962	0.022*	
H12B	0.2573	0.2853	0.3764	0.022*	
C13	0.09600 (19)	0.34550 (16)	0.35520 (16)	0.0168 (5)	
C14	0.0867 (2)	0.39593 (16)	0.46352 (16)	0.0184 (5)	
H14A	0.1635	0.4173	0.4837	0.022*	
C15	0.0199 (2)	0.49927 (16)	0.44477 (17)	0.0227 (5)	
H15A	0.0346	0.5513	0.4989	0.027*	
H15B	−0.0609	0.4854	0.4416	0.027*	
C16	0.0655 (2)	0.53830 (17)	0.33857 (17)	0.0229 (5)	
H16A	0.1135	0.6004	0.3478	0.027*	
H16B	0.0032	0.5574	0.2930	0.027*	
C17	0.1345 (2)	0.44450 (16)	0.29164 (16)	0.0195 (5)	
H17A	0.2148	0.4572	0.3068	0.023*	
C18	−0.0186 (2)	0.30495 (17)	0.31488 (17)	0.0200 (5)	
H18A	−0.0077	0.2683	0.2502	0.030*	
H18B	−0.0687	0.3644	0.3046	0.030*	
H18C	−0.0511	0.2568	0.3647	0.030*	
C19	−0.0274 (2)	0.08996 (18)	0.62344 (17)	0.0235 (5)	
H19A	−0.0211	0.0305	0.5768	0.035*	
H19B	−0.0780	0.1421	0.5946	0.035*	
H19C	−0.0564	0.0657	0.6889	0.035*	
C20	0.1205 (2)	0.43974 (17)	0.17282 (16)	0.0215 (5)	
H20A	0.0400	0.4298	0.1570	0.026*	
C21	0.1867 (2)	0.35143 (18)	0.12036 (17)	0.0233 (5)	

### Atomic displacement parameters (Å^2^)

**Table:**

	*U*^11^	*U*^22^	*U*^33^	*U*^12^	*U*^13^	*U*^23^
O1	0.0291 (10)	0.0234 (8)	0.0217 (8)	0.0045 (7)	−0.0075 (7)	0.0019 (6)
O2	0.0207 (9)	0.0169 (8)	0.0253 (8)	−0.0009 (6)	0.0002 (7)	−0.0033 (7)
O3	0.0265 (10)	0.0221 (8)	0.0284 (9)	0.0012 (7)	0.0011 (7)	0.0086 (7)
O4	0.0228 (10)	0.0311 (9)	0.0322 (9)	0.0030 (7)	0.0013 (8)	−0.0065 (7)
O5	0.0325 (11)	0.0357 (9)	0.0337 (9)	−0.0032 (8)	0.0029 (9)	−0.0137 (8)
O6	0.0277 (11)	0.0355 (10)	0.0323 (9)	0.0005 (8)	0.0001 (8)	−0.0097 (8)
C1	0.0205 (13)	0.0162 (10)	0.0214 (11)	0.0000 (9)	0.0029 (10)	0.0009 (9)
C2	0.0215 (13)	0.0138 (10)	0.0246 (11)	0.0029 (9)	−0.0001 (10)	0.0040 (9)
C3	0.0233 (13)	0.0181 (11)	0.0207 (11)	−0.0007 (9)	0.0016 (10)	0.0040 (9)
C4	0.0266 (14)	0.0222 (11)	0.0184 (11)	0.0017 (10)	0.0032 (10)	0.0001 (9)
C5	0.0213 (13)	0.0206 (11)	0.0184 (11)	0.0007 (9)	0.0022 (10)	0.0021 (9)
C6	0.0282 (14)	0.0233 (11)	0.0207 (11)	0.0111 (10)	0.0036 (10)	−0.0003 (9)
C7	0.0277 (14)	0.0209 (11)	0.0197 (11)	0.0069 (9)	0.0001 (10)	0.0000 (9)
C8	0.0180 (13)	0.0177 (11)	0.0187 (11)	0.0035 (9)	−0.0004 (9)	−0.0006 (9)
C9	0.0155 (12)	0.0173 (10)	0.0174 (11)	0.0011 (9)	0.0009 (9)	0.0009 (9)
C10	0.0188 (13)	0.0180 (10)	0.0184 (11)	0.0023 (9)	−0.0005 (10)	0.0041 (9)
C11	0.0151 (12)	0.0160 (10)	0.0209 (11)	0.0017 (9)	−0.0015 (9)	−0.0020 (9)
C12	0.0176 (13)	0.0186 (10)	0.0181 (11)	0.0013 (9)	0.0010 (10)	−0.0023 (8)
C13	0.0160 (12)	0.0168 (10)	0.0176 (11)	0.0006 (9)	0.0004 (9)	−0.0003 (9)
C14	0.0190 (12)	0.0172 (10)	0.0189 (11)	0.0006 (9)	−0.0028 (9)	−0.0003 (9)
C15	0.0282 (14)	0.0173 (11)	0.0226 (11)	0.0041 (10)	−0.0008 (10)	−0.0001 (9)
C16	0.0290 (14)	0.0164 (10)	0.0232 (12)	0.0019 (10)	−0.0018 (10)	0.0021 (9)
C17	0.0206 (13)	0.0177 (11)	0.0201 (11)	−0.0021 (9)	−0.0018 (10)	0.0030 (9)
C18	0.0183 (13)	0.0194 (11)	0.0222 (11)	0.0019 (9)	−0.0017 (10)	0.0009 (9)
C19	0.0201 (13)	0.0250 (11)	0.0254 (12)	−0.0013 (10)	0.0005 (10)	0.0042 (10)
C20	0.0205 (13)	0.0226 (11)	0.0213 (12)	−0.0019 (9)	−0.0008 (10)	0.0027 (9)
C21	0.0243 (14)	0.0268 (12)	0.0189 (11)	−0.0011 (10)	0.0001 (10)	0.0040 (10)

### Geometric parameters (Å, °)

**Table:**

O1—C3	1.249 (3)	C9—C10	1.581 (3)
O2—C11	1.436 (2)	C9—H9A	0.9800
O2—H2	0.8200	C10—C19	1.554 (3)
O3—C20	1.431 (2)	C11—C12	1.530 (3)
O3—H3	0.8200	C11—H11A	0.9800
O4—C21	1.317 (3)	C12—C13	1.537 (3)
O4—H4	0.8200	C12—H12A	0.9700
O5—C21	1.218 (2)	C12—H12B	0.9700
O6—H61	0.8616	C13—C18	1.535 (3)
O6—H62	0.8572	C13—C14	1.536 (3)
C1—C2	1.331 (2)	C13—C17	1.554 (3)
C1—C10	1.501 (2)	C14—C15	1.535 (3)
C1—H1A	0.9300	C14—H14A	0.9800
C2—C3	1.452 (3)	C15—C16	1.550 (3)
C2—H2A	0.9300	C15—H15A	0.9700
C3—C4	1.458 (3)	C15—H15B	0.9700
C4—C5	1.332 (3)	C16—C17	1.552 (3)
C4—H4A	0.9300	C16—H16A	0.9700
C5—C6	1.510 (3)	C16—H16B	0.9700
C5—C10	1.512 (3)	C17—C20	1.541 (3)
C6—C7	1.525 (3)	C17—H17A	0.9800
C6—H6A	0.9700	C18—H18A	0.9600
C6—H6B	0.9700	C18—H18B	0.9600
C7—C8	1.529 (3)	C18—H18C	0.9600
C7—H7A	0.9700	C19—H19A	0.9600
C7—H7B	0.9700	C19—H19B	0.9600
C8—C14	1.524 (3)	C19—H19C	0.9600
C8—C9	1.553 (3)	C20—C21	1.514 (3)
C8—H8A	0.9800	C20—H20A	0.9800
C9—C11	1.542 (3)		
			
C11—O2—H2	109.5	C11—C12—H12A	108.8
C20—O3—H3	109.5	C13—C12—H12A	108.8
C21—O4—H4	109.7	C11—C12—H12B	108.8
H61—O6—H62	114.6	C13—C12—H12B	108.8
C2—C1—C10	124.04 (13)	H12A—C12—H12B	107.7
C2—C1—H1A	118.0	C18—C13—C14	112.39 (19)
C10—C1—H1A	118.0	C18—C13—C12	111.51 (17)
C1—C2—C3	121.15 (17)	C14—C13—C12	107.16 (17)
C1—C2—H2A	119.4	C18—C13—C17	110.08 (18)
C3—C2—H2A	119.4	C14—C13—C17	99.87 (15)
O1—C3—C2	121.2 (2)	C12—C13—C17	115.30 (18)
O1—C3—C4	121.2 (2)	C8—C14—C15	118.08 (19)
C2—C3—C4	117.6 (2)	C8—C14—C13	114.08 (16)
C5—C4—C3	121.6 (2)	C15—C14—C13	103.91 (17)
C5—C4—H4A	119.2	C8—C14—H14A	106.7
C3—C4—H4A	119.2	C15—C14—H14A	106.7
C4—C5—C6	121.6 (2)	C13—C14—H14A	106.7
C4—C5—C10	123.2 (2)	C14—C15—C16	103.08 (18)
C6—C5—C10	115.21 (18)	C14—C15—H15A	111.1
C5—C6—C7	110.30 (19)	C16—C15—H15A	111.1
C5—C6—H6A	109.6	C14—C15—H15B	111.1
C7—C6—H6A	109.6	C16—C15—H15B	111.1
C5—C6—H6B	109.6	H15A—C15—H15B	109.1
C7—C6—H6B	109.6	C15—C16—C17	106.71 (17)
H6A—C6—H6B	108.1	C15—C16—H16A	110.4
C6—C7—C8	112.93 (17)	C17—C16—H16A	110.4
C6—C7—H7A	109.0	C15—C16—H16B	110.4
C8—C7—H7A	109.0	C17—C16—H16B	110.4
C6—C7—H7B	109.0	H16A—C16—H16B	108.6
C8—C7—H7B	109.0	C20—C17—C16	111.11 (18)
H7A—C7—H7B	107.8	C20—C17—C13	117.47 (18)
C14—C8—C7	111.05 (17)	C16—C17—C13	104.20 (16)
C14—C8—C9	108.23 (18)	C20—C17—H17A	107.9
C7—C8—C9	109.56 (17)	C16—C17—H17A	107.9
C14—C8—H8A	109.3	C13—C17—H17A	107.9
C7—C8—H8A	109.3	C13—C18—H18A	109.5
C9—C8—H8A	109.3	C13—C18—H18B	109.5
C11—C9—C8	114.68 (17)	H18A—C18—H18B	109.5
C11—C9—C10	114.52 (16)	C13—C18—H18C	109.5
C8—C9—C10	111.82 (17)	H18A—C18—H18C	109.5
C11—C9—H9A	104.8	H18B—C18—H18C	109.5
C8—C9—H9A	104.8	C10—C19—H19A	109.5
C10—C9—H9A	104.8	C10—C19—H19B	109.5
C1—C10—C5	112.22 (16)	H19A—C19—H19B	109.5
C1—C10—C19	105.45 (15)	C10—C19—H19C	109.5
C5—C10—C19	108.68 (18)	H19A—C19—H19C	109.5
C1—C10—C9	110.70 (16)	H19B—C19—H19C	109.5
C5—C10—C9	105.37 (16)	O3—C20—C21	107.00 (16)
C19—C10—C9	114.56 (18)	O3—C20—C17	109.98 (17)
O2—C11—C12	110.45 (16)	C21—C20—C17	114.63 (19)
O2—C11—C9	111.34 (16)	O3—C20—H20A	108.4
C12—C11—C9	113.44 (16)	C21—C20—H20A	108.4
O2—C11—H11A	107.1	C17—C20—H20A	108.4
C12—C11—H11A	107.1	O5—C21—O4	123.4 (2)
C9—C11—H11A	107.1	O5—C21—C20	123.0 (2)
C11—C12—C13	113.65 (17)	O4—C21—C20	113.57 (18)
			
C10—C1—C2—C3	−3.6 (3)	O2—C11—C12—C13	77.4 (2)
C1—C2—C3—O1	−174.30 (19)	C9—C11—C12—C13	−48.4 (3)
C1—C2—C3—C4	4.9 (3)	C11—C12—C13—C18	−68.5 (2)
O1—C3—C4—C5	176.5 (2)	C11—C12—C13—C14	54.9 (2)
C2—C3—C4—C5	−2.7 (3)	C11—C12—C13—C17	165.03 (17)
C3—C4—C5—C6	−177.9 (2)	C7—C8—C14—C15	−58.4 (3)
C3—C4—C5—C10	−0.9 (4)	C9—C8—C14—C15	−178.66 (18)
C4—C5—C6—C7	120.0 (2)	C7—C8—C14—C13	179.2 (2)
C10—C5—C6—C7	−57.2 (3)	C9—C8—C14—C13	58.9 (2)
C5—C6—C7—C8	52.4 (3)	C18—C13—C14—C8	60.7 (2)
C6—C7—C8—C14	−173.2 (2)	C12—C13—C14—C8	−62.1 (2)
C6—C7—C8—C9	−53.7 (3)	C17—C13—C14—C8	177.38 (19)
C14—C8—C9—C11	−48.7 (2)	C18—C13—C14—C15	−69.2 (2)
C7—C8—C9—C11	−169.93 (19)	C12—C13—C14—C15	167.97 (17)
C14—C8—C9—C10	178.76 (17)	C17—C13—C14—C15	47.5 (2)
C7—C8—C9—C10	57.5 (2)	C8—C14—C15—C16	−164.81 (19)
C2—C1—C10—C5	0.1 (2)	C13—C14—C15—C16	−37.3 (2)
C2—C1—C10—C19	−118.10 (18)	C14—C15—C16—C17	12.2 (2)
C2—C1—C10—C9	117.47 (18)	C15—C16—C17—C20	144.16 (19)
C4—C5—C10—C1	2.2 (3)	C15—C16—C17—C13	16.7 (2)
C6—C5—C10—C1	179.43 (17)	C18—C13—C17—C20	−43.8 (3)
C4—C5—C10—C19	118.5 (2)	C14—C13—C17—C20	−162.2 (2)
C6—C5—C10—C19	−64.4 (2)	C12—C13—C17—C20	83.4 (3)
C4—C5—C10—C9	−118.3 (2)	C18—C13—C17—C16	79.6 (2)
C6—C5—C10—C9	58.9 (2)	C14—C13—C17—C16	−38.8 (2)
C11—C9—C10—C1	47.5 (2)	C12—C13—C17—C16	−153.20 (19)
C8—C9—C10—C1	−179.92 (15)	C16—C17—C20—O3	58.4 (2)
C11—C9—C10—C5	169.00 (19)	C13—C17—C20—O3	178.26 (17)
C8—C9—C10—C5	−58.4 (2)	C16—C17—C20—C21	179.04 (18)
C11—C9—C10—C19	−71.6 (2)	C13—C17—C20—C21	−61.2 (3)
C8—C9—C10—C19	61.0 (2)	O3—C20—C21—O5	−120.4 (2)
C8—C9—C11—O2	−80.3 (2)	C17—C20—C21—O5	117.4 (2)
C10—C9—C11—O2	51.0 (2)	O3—C20—C21—O4	58.8 (2)
C8—C9—C11—C12	45.0 (3)	C17—C20—C21—O4	−63.5 (2)
C10—C9—C11—C12	176.29 (18)		

### Hydrogen-bond geometry (Å, °)

**Table:**

*D*—H···*A*	*D*—H	H···*A*	*D*···*A*	*D*—H···*A*
O2—H2···O1^i^	0.82	1.92	2.708 (2)	161
O3—H3···O2^ii^	0.82	2.06	2.819 (2)	153
O4—H4···O6^iii^	0.82	1.84	2.646 (2)	167
O6—H61···O1	0.86	1.92	2.765 (2)	165
O6—H62···O5^iv^	0.86	2.10	2.938 (2)	166

Symmetry codes: (i) −*x*+1/2, −*y*, *z*−1/2; (ii) −*x*, *y*+1/2, −*z*+1/2; (iii) *x*, *y*, *z*−1; (iv) *x*+1/2, −*y*+1/2, −*z*+1.
